# A Case of Progressive Dyspnea: Lymphocytic Interstitial Pneumonia in Collagen Vascular Disease

**DOI:** 10.7759/cureus.18427

**Published:** 2021-10-01

**Authors:** Jonathan Moore, Akhilesh Mahajan, Sravani Gajjala, Priyanka Makkar

**Affiliations:** 1 Pulmonary and Critical Care, Lenox Hill Hospital, New York, USA; 2 Internal Medicine, Lenox Hill Hospital, New York, USA

**Keywords:** lymphocytic interstitial pneumonia, cystic lung disease, chronic hypoxic respiratory failure, mixed connective tissue disease, sjogren's disease, anti-ku inflammatory myopathy

## Abstract

We describe an interesting rare case of a 61-year-old woman who was admitted to our hospital for exertional dyspnea, non-productive cough, and generalized weakness of six months duration. Her computed tomography was significant for ground-glass opacities combined with bibasilar consolidations and numerous pulmonary cysts. There can be a significant overlap in imaging findings of post-coronavirus disease 2019 (COVID-19) lung disease and interstitial lung disease from autoimmune diseases. We review in extensive detail the differential diagnosis for these imaging findings from a pulmonologist’s perspective and discuss investigations required for further workup. Our patient underwent transbronchial biopsy and was eventually diagnosed with lymphocytic interstitial pneumonia with Sjogren predominant mixed connective tissue disease. We also review in detail the current literature and prognosis for this interesting disease.

## Introduction

Sjogren’s syndrome is a commonly encountered autoimmune disorder with a worldwide distribution. The syndrome is characterized by lymphocytic infiltration of exocrine tissues resulting in the typical sicca symptoms [1]. It can occur with other collagen vascular disorders and when it does it is termed secondary Sjogren’s syndrome. There is a predilection for the female sex. Criteria have been proposed for the classification of Sjogren’s syndrome by the American College of Rheumatology and the European League Against Rheumatism. Our patient had a high anti-Sjogren's syndrome-related antigen A (anti-SSA) titer and signs of xerostomia.

Extra-glandular symptoms of Sjogren’s disease can involve almost every organ system and include polyarthralgia, erosive arthritis, peripheral neuropathies, oligoclonal and monoclonal gammopathies, cryoglobulinemia, and vasculitis [2]. Pulmonary involvement can be seen in 10-20% of patients and disease activity is seen primarily in the airways and interstitium - pleural disease and pulmonary hypertension are extremely uncommon [3]. Symptoms are variable in patients and may range from mild dyspnea to severe disability. Pulmonary function testing commonly shows restrictive lung disease. Airway involvement in these patients may manifest as bronchial hyperresponsiveness (42-60%), bronchiolitis (12-14%) - predominantly follicular, and cylindrical bronchiectasis (7-54%) [3]. We describe a case of lymphocytic interstitial pneumonia with Sjogren predominant mixed connective tissue disorder (MCTD) and complicated by anti-Ku positive inflammatory myopathy.

## Case presentation

**Figure 1:**
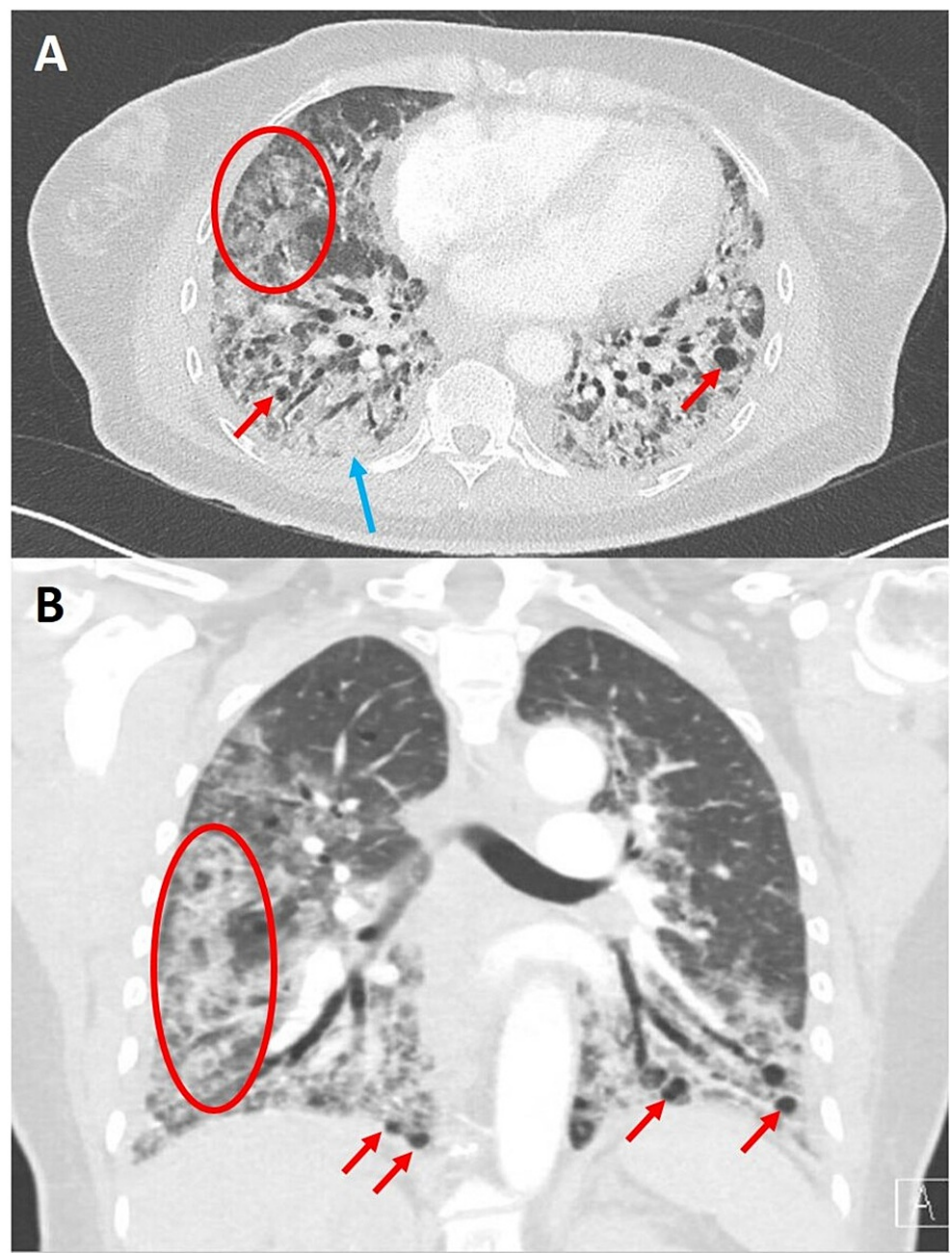
Representative axial (A) and coronal (B) CT angiography performed during patient's admission for COVID-19 pneumonia. Noted are diffuse ground-glass opacities (red circle), bibasilar consolidations (blue arrow), and dilated airways and scattered cystic structures (red arrow). COVID-19, coronavirus disease 2019.

A 61-year-old woman presented to the hospital with an eight-day history of chest pressure, exertional dyspnea, palpitations, non-productive cough, and generalized weakness. She was previously diagnosed with community-acquired pneumonia and treated with antibiotics without improvement. The patient reported a history of hypertension, unintentional weight loss, gastric reflux, and coronavirus disease 2019 (COVID-19) pneumonia six months prior to presentation. During COVID-19, she required admission to the hospital for acute hypoxic respiratory failure and completed a course of remdesivir and steroids. Computed tomography angiography (CTA) during her admission demonstrated diffuse ground-glass opacities, bibasilar consolidations with air bronchograms, mild centrilobular emphysema, scattered small cystic structures, and absence of pulmonary emboli (Figure 1). She was discharged home on supplemental oxygen at that time. Vital signs on admission were significant for a temperature of 36.8°C, heart rate of 100 beats per minute, blood pressure of 128/88 mmHg, respiratory rate of 27 breaths per minute, and oxygen saturation was 90% on 3 liters nasal cannula. Physical examination showed a frail appearing elderly female with poor dentition and multiple oral white plaques, which could be scraped off. Lung auscultation demonstrated diminished breath sounds and fine inspiratory crackles in bilateral posterior lung fields. She exhibited symmetric lower extremity motor weakness. Her results were significant for a hemoglobin level of 7.9 g/dL, serum gamma protein gap of 6.3 g/dL, C-reactive protein of 3.75 mg/dL, lactate dehydrogenase (LDH) of 756 U/L, and serum ferritin of 1,568 ng/mL.

**Figure 2:**
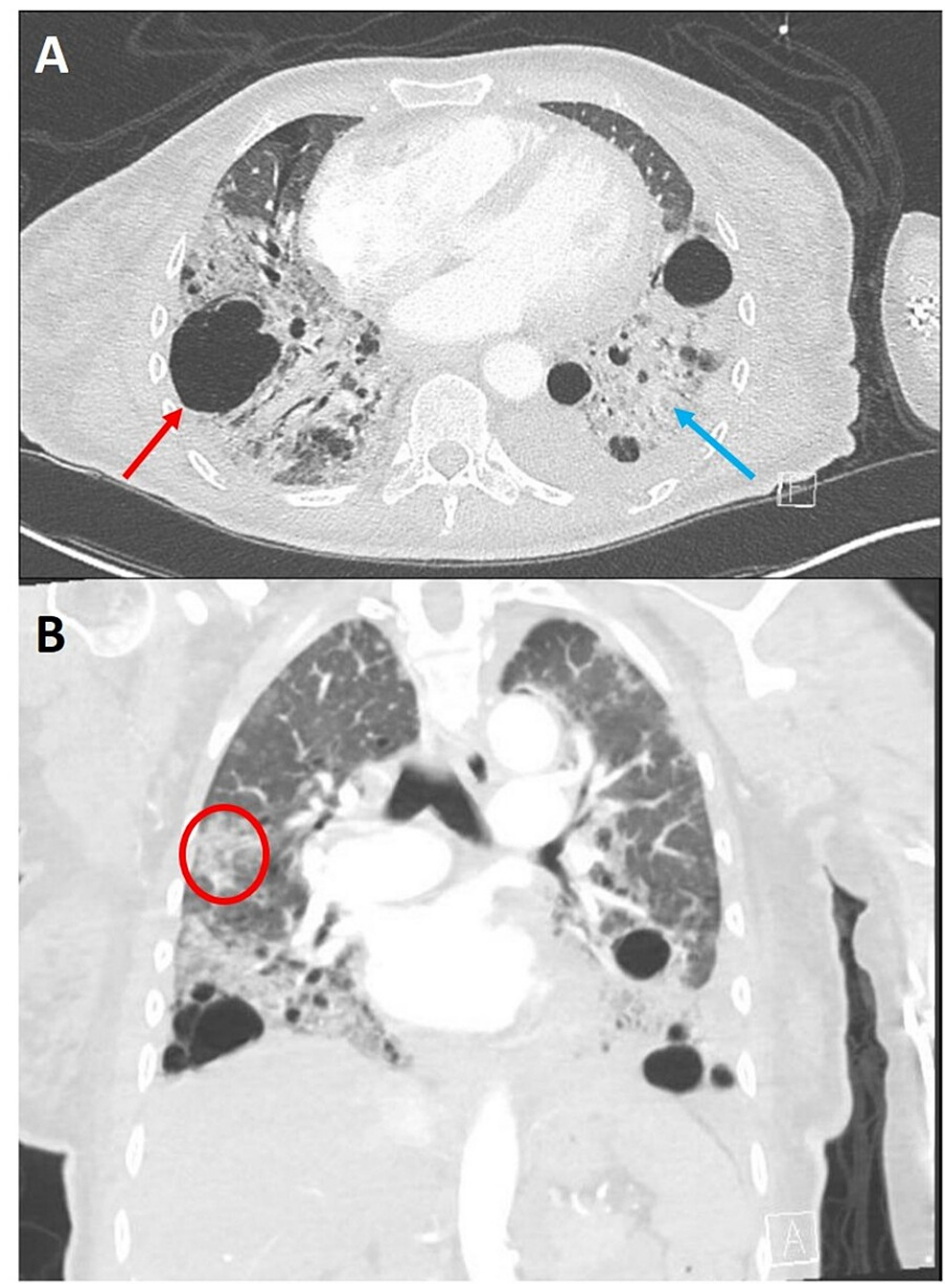
Representative axial (A) and coronal (B) CT angiography images of patient’s current admission. Axial images demonstrate interval progression of heterogenous cysts (red arrow), some of which appear to have coalesced into bullae. Cysts appear to be distributed peripherally. Coronal cuts demonstrate a craniocaudal gradient. Also seen are ground-glass opacities (red circle), dense consolidations (blue arrow), a left-sided pleural effusion, and air bronchograms.

An anteroposterior chest X-ray demonstrated diffuse interstitial infiltrates, blunting of bilateral costophrenic angles, and evidence of hypo lucent structures at the lung bases. The CTA of the chest showed diffuse peripheral consolidations with possible subpleural sparing, interval worsening of cystic structures with a basilar predominance, bilateral ground-glass opacities distributed in the upper lung zones, small left pleural effusion, a dilated main pulmonary artery, and the absence of pulmonary emboli (Figure 2). The patient underwent bronchoscopy with transbronchial biopsy of the right upper lobe with pathology showing interstitial edema and mild fibrosis. This was interpreted as non-diagnostic for specific pulmonary pathology.

Workup for connective tissue diseases (CTD) was performed and it revealed positive antinuclear antibodies with a speckled pattern and titers of 1:320. Other pertinent laboratory data included elevated rheumatoid factor levels of 60 IU/mL, double-stranded DNA antibody level of 209 IU/mL, and positive atypical anti-neutrophil cytoplasmic antibody (ANCA), and Sjogren’s antibody testing demonstrated the presence of anti-SS-A (Ro) antibodies (8 antibody index). Quantitative testing of anti-immunoglobulin G (IgG) antibodies against SS-A revealed a level of 211 units (upper limit of normally less than 20 units). Given the patient’s proximal muscle weakness and elevated serum creatine kinase (2855 U/L), an inflammatory myomarker panel was checked. It demonstrated the presence of an anti-Ku antibody. Serum immunofixation was performed which showed a polyclonal expansion and an IgG kappa band.

A diagnosis of lymphocytic interstitial pneumonia with Sjogren predominant MCTD and anti-Ku positive inflammatory myopathy was made based on the patient's chest CT imaging and immunological panel. The patient was started on high-dose systemic corticosteroids (1 mg/kg) during her hospitalization with improvement in her hypoxic respiratory failure. After initiation of her corticosteroids, her strength improved over the next two to three weeks, and with physical therapy, she was able to return to her prehospitalization functional status. The patient was discharged with plans to follow clinically, and outpatient consider initiation of rituximab.

## Discussion

Radiologic differential discussion

A pulmonary cyst is defined radiographically as round parenchymal lucency with thin walls and a well-defined interface with the surrounding lung parenchyma. Cysts can present with different characteristic patterns, which help narrow the differential diagnosis. Differential for cystic lung disease includes lymphocytic interstitial pneumonia (LIP), desquamative interstitial pneumonia (DIP), cystic lung light chain deposition disease (CL-LCDD), pneumatoceles, or infectious processes. This patient presents with interval worsening of cystic lung disease over the course of six months.

Lymphocytic Interstitial Pneumonia

LIP is an uncommon interstitial pneumonia with a variable clinical course. The most frequently described radiographic appearance on high resolution computed tomography (HRCT) is that of diffuse, multifocal cysts with small nodules. Thickening of the bronchovascular bundles, interlobular septal thickening, and lymphadenopathy can be seen. Large nodules, pleural effusions, and consolidations are atypical of LIP. Histology demonstrates diffuse hyperplasia of the bronchus-related lymphoid tissues with polyclonal lymphoid infiltrate. It is most frequently described in Sjogren’s disease but can be associated with systemic lupus erythematosus, rheumatoid arthritis, dysproteinemia syndromes, hypogammaglobulinemia, and multiple viral infections [4]. LIP can be the first presenting sign of rheumatic diseases.

Desquamative Interstitial Pneumonia

DIP is a rare interstitial pneumonia found predominantly in active smokers. DIP has been described in patients with environmental exposures to inorganic particles, but a causal relationship is unclear. The progression of DIP is typically insidious but may rarely be fulminant. DIP may be seen in autoimmune collagen vascular diseases. The radiographic appearance on HRCT is not fully characterized. Infiltrates are typically diffuse ground-glass opacities with a lower lung predominance; the infiltrates may also have a peripheral distribution. Cystic lung changes may be seen, however, these are not typical.

Cystic Lung - Light Chain Deposition Disease and Amyloidosis

Light chain deposition disease (LCDD) is a syndrome in which immunoglobulin light chains are deposited in amorphous strands leading to end-organ damage. LCDD is associated with plasma cell dyscrasias or lymphoproliferative diseases, which produce excess light chains. Pulmonary involvement due to LCDD is rare. The radiographic description is limited to small retrospective series but virtually all cases demonstrate evidence of cystic lung disease with a lower lung predominance and randomly distributed cysts [5].

Amyloidosis is a systemic disease that can affect every organ system. Pulmonary involvement may show nodules, tracheobronchial infiltration, pleural effusions, cystic lung changes, pulmonary hypertension, and involvement of the upper airways [6]. Primary pulmonary amyloidosis is a rare clinical entity but has been reported in patients with primary Sjogren’s syndrome [7].

Infectious Causes

Pneumatoceles are thin-walled, gas-filled structures within the lung parenchyma. They are the result of injury or inflammation to a bronchiole resulting in a check valve mechanism for gas entry and air trapping. Any pneumonia may result in the formation of pneumatoceles. They typically present during active infection and tend to regress as pneumonia resolves. COVID-19 pneumonia has also been shown to result in the formation of pneumatoceles but there is little evidence regarding their long-term outcomes after acute illness.

Clinical discussion

Historically, LIP was the most common interstitial lung disease associated with primary Sjogren’s syndrome [8]. Newer data have shown that the most common subtype of interstitial lung disease (ILD) is nonspecific interstitial pneumonia (NSIP) with an estimated prevalence of 28-60%. LIP and usual interstitial pneumonia (UIP) each can be seen in approximately 20% of Sjogren’s syndrome-related ILD [8]. Approximately 10% of Sjogren’s associated ILD can show evidence of cystic changes, which occur in conjunction with LIP, amyloidosis, lymphoma, and LCDD. Patients with elevated antinuclear antibody (ANA), rheumatoid factors, and serologic markers of inflammation are more prone to the development of ILD.

A traditional transbronchial forceps biopsy was performed but was non-diagnostic. The patient had a pre-existing supplemental oxygen requirement with diffuse pulmonary involvement, and a surgical lung biopsy in this patient would confer increased morbidity and mortality without clinical benefit. Due to post-operative morbidity and mortality, individuals with a diagnosed CTD and radiographic evidence of a congruent ILD may be diagnosed clinically and do not require pathologic diagnosis prior to initiating treatment. Given her radiographic findings and Sjogren’s disease, a unifying diagnosis of LIP in this patient is likely.

The patient’s case was further complicated by an autoimmune myositis with a positive anti-Ku antibody. Anti-Ku is a non-specific autoantibody that has been detected in several CTD including systemic sclerosis, systemic lupus erythematosus (SLE), idiopathic inflammatory myopathy, Sjogren’s disease, and mixed CTD [9]. Elevated creatine kinase in patients with anti-Ku has been shown to increase the risk of developing ILD 22-fold. Patients with ILD and anti-Ku tended to be Hispanic, older at diagnosis, and had fewer cutaneous manifestations of their collagen vascular diseases [10]. Those who develop ILD can have higher rates of corticosteroid-resistant ILD, necessitating the use of immunosuppressive agents.

The natural history and prognosis of LIP are poorly understood. Treatment of LIP with corticosteroids initially may stabilize lung function and prevent progression of ILD [11]. Azathioprine, cyclophosphamide, and rituximab have been utilized for refractory cases and as a steroid-sparing therapy in some patients with LIP [12]. In a small series of patients, the median survival time from diagnosis of LIP was 11.5 years although other studies quote a five-year survival rate of 30-50% [11]. Malignant transformation to lymphoma has been described in 5% of patients with LIP [11]. Clinical trials are lacking due to the low incidence of patients with LIP and there is no consensus regarding its treatment.

## Conclusions

In summary, we present a rare case of interstitial lung disease as the presenting symptom of a mixed connective tissue disorder accompanied by an inflammatory myopathy. Prior to COVID-19, the patient had no symptoms of a connective tissue disorder and had been in good health. Subsequent to her COVID-19 infection, she experienced rapid progression of her cystic interstitial lung disease leading ultimately to her diagnosis of lymphocytic interstitial pneumonia. Since the onset of the COVID-19 pandemic, interstitial abnormalities are common findings on chest CT. However, familiarity with the differential of these findings is important as they may be an early marker of a collagen vascular disease. Lymphocytic interstitial pneumonia is a rare clinical entity and there are no guidelines regarding its treatment or prognosis. Clinical trials and prospective research are needed to guide future therapy.
